# Nairobi Newborn Study: a protocol for an observational study to estimate the gaps in provision and quality of inpatient newborn care in Nairobi City County, Kenya

**DOI:** 10.1136/bmjopen-2016-012448

**Published:** 2016-12-21

**Authors:** Georgina A V Murphy, David Gathara, Jalemba Aluvaala, Jacintah Mwachiro, Nancy Abuya, Paul Ouma, Robert W Snow, Mike English

**Affiliations:** 1 Nuffield Department of Medicine, Centre for Tropical Medicine and Global Health, University of Oxford, Oxford, UK; 2 Kenya Medical Research Institute/Wellcome Trust Research Programme, Nairobi, Kenya; 3 Department of Paediatrics and Child Health, College of Health Sciences, University of Nairobi, Nairobi, Kenya; 4 Nairobi City County Government, Nairobi, Kenya

**Keywords:** Newborns, EPIDEMIOLOGY, Africa

## Abstract

**Introduction:**

Progress has been made in Kenya towards reducing child mortality as part of efforts aligned with the fourth Millennium Development Goal. However, little advancement has been made in reducing mortality among newborns, which now accounts for 45% of all child deaths. The frequently unanticipated nature of neonatal illness, its severity and the high dependency of sick newborns on skilled care make the provision of inpatient hospital services one key component of strategies to improve newborn survival.

**Methods and analyses:**

This project aims to assess the availability and quality of inpatient newborn care in hospitals in Nairobi City County across the public, private and not-for-profit sectors and align this to the estimated need for such services, providing a description of the quantity and quality gaps between capacity and demand. The population level burden of disease will be estimated using morbidity incidence estimates from a literature review applied to subcounty estimates of population-adjusted births, providing a spatially disaggregated estimate of need within the county. This will be followed by a survey of neonatal services across all health facilities providing 24/7 inpatient newborn care in the county. The survey will include: a retrospective audit of admission registers to estimate the usage of facilities and case-mix of patients; a structural assessment of facilities to gain insight into capacity; a questionnaire to nursing staff focusing on the process of delivering key obstetric and neonatal interventions; and a retrospective case audit to assess adherence to guidelines by clinicians.

**Ethics and dissemination:**

This study has been approved by the Kenya Medical Research Institute Scientific and Ethics Review Unit (SSC protocol No.2999). Results will be disseminated: to participating facilities through individualised reports and a joint workshop; to local and national stakeholders through meetings and a summary report; and to the international community through peer-review publication and international meetings.

**Strengths and limitation of this study**

- New approaches to evaluating the provision and quality of services for small and sick newborns have been developed. The methods and data collection tools are applicable and available for use in other low-resource settings.
- Stakeholders and policymakers are engaged throughout this project to ensure that the findings are meaningful to those creating policy and aiming to improve practice.
- The estimation of population-level burden of disease is reliant on these data being available in the literature, as local statistics are not available and direct estimation is outside the remit of this study. Such literature is limited, which will, in turn, limit our ability to estimate the gap between need and availability of services.
- Our ability to estimate the usage and case-mix of facilities and the quality of care delivered by clinicians will be limited by the availability and quality of data recorded on the registers and records in facilities.
- This complex observational study will provide valuable input to the global effort to improve data on service delivery for sick newborns. However, high-quality local routine surveillance systems are necessary to ensure ongoing affordable monitoring of the quality and provision of inpatient newborn care.

## Introduction

Globally, substantial progress has been made in reducing child mortality, declining from 12.7 million deaths in 1990 to 5.9 million in 2015.^1^ However, the majority of countries in Africa did not make sufficient progress to meet their fourth Millennium Development Goal targets to reduce child deaths by two-thirds between 1990 and 2015. In part, this is because there has been little reduction in neonatal mortality (deaths in the first 28 days of life). Consequently, neonatal mortality now accounts for 45% of all child mortality in many of these countries.^2^ In order to make continued improvements in child survival in the new era of Sustainable Development Goals, significant emphasis on, and progress in, reducing neonatal mortality will be needed.^3^

The major causes of neonatal mortality and long-term morbidity include: preterm birth; intrauterine growth restriction; intrapartum-related neonatal encephalopathy and infections.^4^ Much of the mortality and long-term morbidity associated with these conditions is preventable with cost-effective interventions and the provision of universal access to basic but high-quality health services.^5^ ^6^ The provision of basic and comprehensive emergency obstetric and newborn care (EmONC), with resuscitation at birth for babies that require it, is of vital importance for disease prevention and reduction in intrapartum mortality.^7^ ^8^ In addition, the management of neonatal conditions through facility-based inpatient interventions, such as phototherapy for jaundice, intravenous antibiotics for sepsis, assisted feeding for premature newborns and oxygen for respiratory distress syndrome, has been shown to dramatically reduce neonatal mortality.^7^ ^8^

While international and national policies recognise the need to create demand for, and supply of, skilled delivery and maternal health facilities, rather less attention has been paid to the provision of high-quality care for high-risk or sick newborns in low-resource settings.^6^ ^9^ In Nairobi City County, Kenya, an estimated 88.7% of births take place within health facilities, compared with 61.2% on a national level.^10^ Yet, the neonatal mortality rate in Nairobi is considerably higher than elsewhere in Kenya (39 per 1000 compared with 19–25 per 1000 live births).^10^ Many of the mothers deliver in small facilities that are poorly equipped to provide appropriate care to a severely ill newborn. Even where sick newborns access hospital care, data from Kenyan public hospitals would suggest that the quality of care is often poor.^11^

In order to plan improvements in the equitable provision of care, it is first necessary to better understand the provision, capacity, quality and accessibility of existing newborn services and how this compares to the need for such services.^4^ ^12^ Under an ideal system, disease burden can be derived from routine health information systems that provide comprehensive data from vital registration, health facility usage and cause of death notification.^12^ However, in settings where such information systems are weak (including Kenya^13^), disease burdens must often be estimated from limited epidemiological studies of incidence. On service delivery, despite increased national efforts to characterise health facilities through initiatives such as the Kenyan Master Facility List and the 2013 Service Availability and Readiness Assessment Mapping (SARAM), knowledge on which services are provided, where, by whom and of what quality remains very limited. Furthermore, SARAM collects very little data on inpatient newborn care. In the short term, characterising the burden-to-service availability demands alternative approaches of data assembly to provide a more informed platform to understand the provision of accessible high-quality care.

Insight into the characteristics and distribution of neonatal services and the distribution of incidence of neonatal morbidity will be important to inform the design of possible interventions to improve the provision and quality of, and access to, facility-based newborn care in Kenya. This work, while being carried out in Nairobi, will also develop methods of interest nationally and internationally as Kenya and other low-resource settings develop longer term plans for enhanced service provision.

### Aims and objectives

The overall aim of this project is to quantify and characterise the supply of facility-based inpatient newborn care, in terms of capacity, quality and accessibility; to estimate the expected demand for inpatient neonatal care in Nairobi City County; and to guide strategy on addressing any gaps identified.

The objectives of the study are to: (1) estimate the magnitude and distribution of the burden of expected neonatal morbidity in Nairobi City County; (2) map the location of existing facilities capable of providing inpatient newborn care; (3) estimate the usage of facilities providing inpatient newborn care, including profiling the case-mix of neonatal morbidities treated within these facilities; (4) assess the quality of EmONC and inpatient newborn care provided in terms of structure and process; and (5) describe any shortfall between the expected incidence of disease (burden) and the capacity for caring for sick neonates (supply).

### Global public health relevance

This research will be conducted in partnership with county and national policymakers as part of efforts to provide evidence for long-term policy on service provision. An expert advisory group, including partners from the Ministry of Health, Nursing Council of Kenya, University of Nairobi and Nairobi City County, will support this study. This expert group will regularly meet at the beginning, in the middle and at the end of the study to discuss plans, progress and dissemination of information, respectively. Such an approach to stakeholder engagement aims to ensure that the outputs of the study are of direct relevance and use to local policymakers and practitioners who strive to improve the care for sick newborns and reduce neonatal mortality in this low-resource setting.

In providing this protocol by publication and the data collection tools by request, we hope to encourage others to consider how evidence on service provision for sick newborns may be gathered and used both globally and locally. The study highlights the current lack of information available to make policy and health service strengthening decisions in Kenya, as in many low-income and middle-income countries. However, it also demonstrates how the available information from the literature, admission registers and medical records, complemented by standardised data collection tools, may be used to identify key gaps in service provision and quality and to inform decision-making. We encourage replication of this study in other locations in a global effort to reduce neonatal mortality.

## Methods and analyses

### Study design and procedures

**Figure 1:**
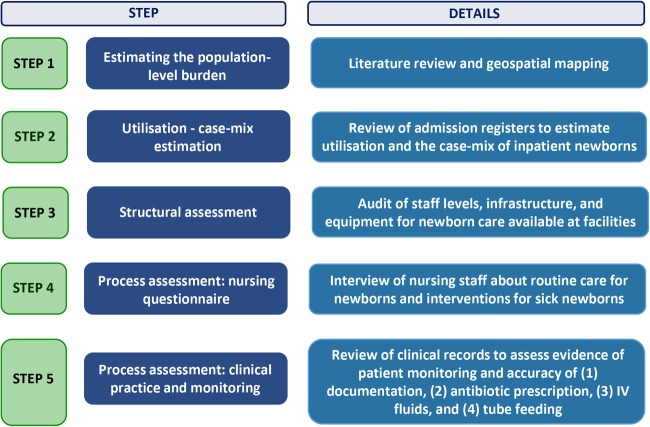
Overview of study procedures.

This study is primarily cross-sectional descriptive in design, drawing on a variety of methods including health services assessment, retrospective case audit and survey questionnaire. Literature review is also conducted as part of this study. The steps of the Nairobi Newborn Study are outlined in figure 1.

**Figure 2:**
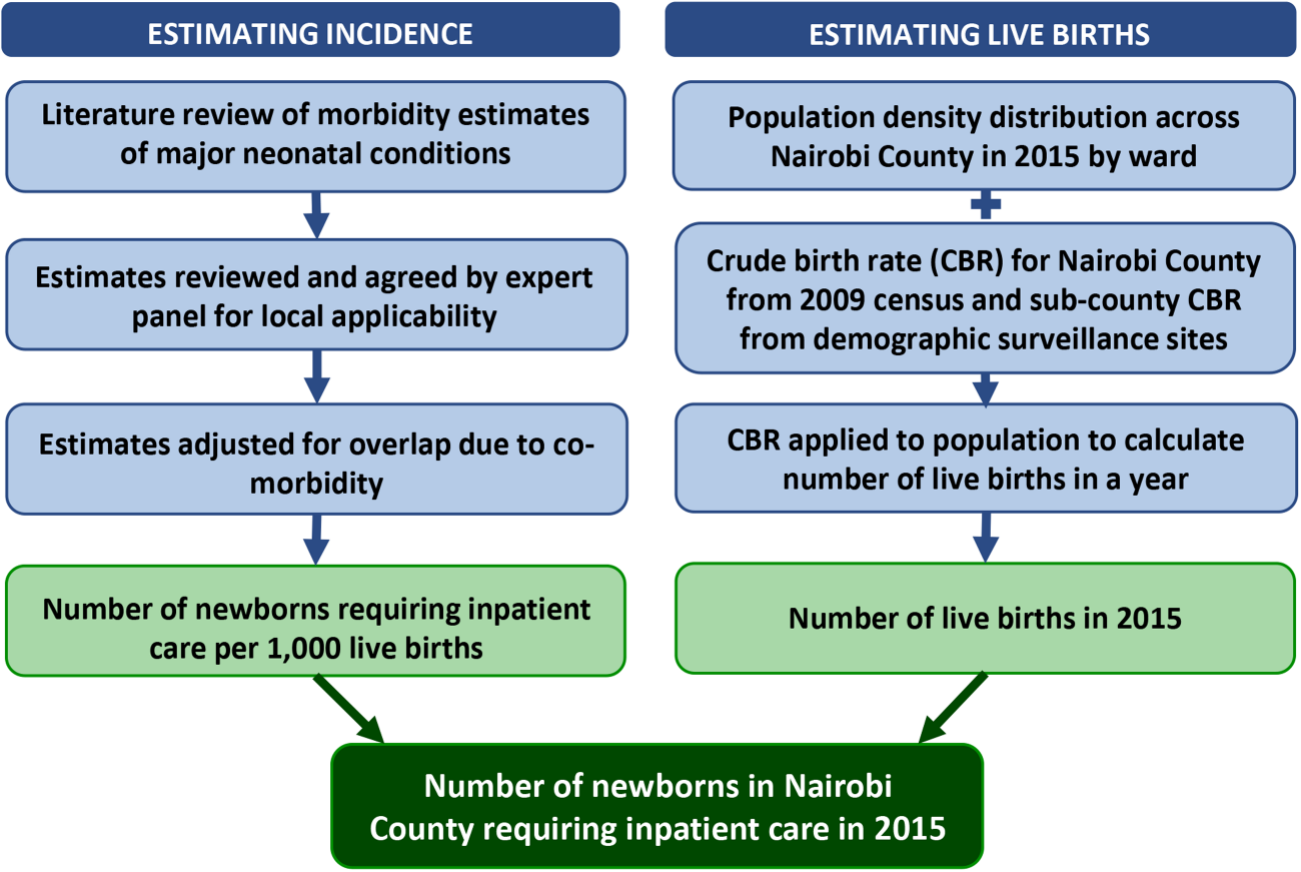
Estimating the need for neonatal services (study step 1).

We will begin with estimating the population-level burden of neonatal conditions using morbidity incidence estimates from the literature review (figure 1, step 1). The available literature will be discussed with the expert group in order to decide on the incidence estimates that are most applicable to the Nairobi City County. Based on these findings, estimates from the literature will be combined (accounting for comorbidities/overlapping estimates where possible) to calculate the anticipated need for neonatal services. Combinations of subcounty population census data at varying spatial resolutions will be used to distribute the total population across the County boundaries. The units of interest will be wards.^14^ Wards are the lowest level of decision-making for the County and the lowest spatial scale for which variations in social inequalities are defined by the national government.^14^ ^15^ Overall crude birth rates for Nairobi County, derived from the 2009 national census, will be compared with subcounty, special group estimates available from demographic surveillance sites within Nairobi.^10^ ^16^ ^17^ Adjusted estimates of new births within the County will be used to generate spatially representative denominators within the County. This process is summarised in figure 2.

Empirical data collection will focus on quantifying the availability and quality of inpatient newborn services across all facilities in Nairobi City County that provide care for sick newborns 24 hours a day for 7 days a week (24/7). Facilities that might be eligible will initially be identified using the Kenya Master Facility list.^18^ ^19^ This provisional list of eligible facilities will then be discussed with local obstetricians and paediatricians to exclude ineligible facilities and add missing ones. Facilities will be telephoned to evaluate their eligibility and finally visited by the research team to confirm eligibility. During this visit, advice will also be sought from facility staff on additional facilities that might be missed from our list. Data collection will be conducted in four steps (figure 1, steps 2–5).

A retrospective audit of maternal and newborn admission registers will be conducted to estimate the usage of facilities and case-mix of neonatal patients (figure 1, step 2). Data from registers will be entered onsite in facilities by experienced and trained data clerks into a purpose-designed standardised data capture tool created in Research Electronic Data Capture (REDCap). This data capture tool will incorporate inbuilt data checks and predesigned cleaning scripts will be run daily and weekly to ensure data integrity. Where admission registers are not available, the admission information will be obtained from the patient medical records.

A structural assessment of facilities will be performed in the maternity and neonatal units to gain insight into staffing, infrastructure and equipment capacity (figure 1, step 3). These data will be collected through direct observation by assistant research officers (clinically trained), recorded on a paper-based structured survey tool, and double data entered in a purpose-designed standardised REDCap tool.

A knowledge questionnaire will be administered to nursing staff on duty in the maternity and neonatal units focusing on the process of delivering key obstetric and neonatal interventions (figure 1, step 4). Questions will include clinical scenarios where nurses are asked the steps that they would take (eg, keeping the newborn warm and newborn resuscitation), direct questions about clinical care guidelines (eg, cord clamping timing and baby checks during phototherapy treatment), and their opinions on availability of essential equipment and drugs (eg, intravenous fluids and oxygen) and frequency with which key newborn interventions are performed in the facility (eg, cleaning the cord with chlorhexidine digluconate and continuous positive airway pressure (CPAP) for respiratory distress in preterm newborns). The questionnaire will be conducted as a face-to-face interview by trained research assistants, who will read aloud questions and enter answers into a preprogrammed and custom-designed REDCap survey tool. Care will be taken to ensure minimal disruption to nurses while on duty and hence their care to patients. Interviews will be conducted during the nurse's break (refreshments will be provided) or at the end of their shift.

Finally, we will conduct a retrospective case audit of neonatal inpatient records to assess adherence to national guidelines by clinicians and documented evidence of patient monitoring (figure 1, step 5). Information of interest to be captured includes recording of patient signs and symptoms, diagnosis of patient, treatments and investigations prescribed, antibiotic dosage (by patient weight) prescribed, and dose and route of oxygen, fluids, and feeds prescribed. The same process as for entering data for admission registers will be applied. Where data are missing from records, data clerks will actively record these as missing to ensure that the adequacy of documentation can be quantified.

The expected shortfall in supply to meet demand will be geospatially mapped in order to provide insight into the areas of the county with the greatest need for improved access to care. All data collection tools and standard operating procedures are available on request.

### Setting and participants

In Nairobi City County, over half the population of 3.14 million people are estimated to live in low-income areas,^20^ inequality is massive and estimated neonatal mortality is considerably higher than the national average.^10^ Data collection for this study will take place in facilities within Nairobi City County that provide 24/7 inpatient newborn care. These facilities will be identified using the Kenyan Master Facility List and advice from local paediatricians and obstetricians.^18^

### Sampling

All eligible facilities will be invited to participate in the study. Information does not currently exist on the number of facilities providing inpatient newborn services in Nairobi or the capacity of facilities to provide this care. However, in consultation with local experts, we anticipate ∼30 facilities to be eligible. Step 1 of this study is a literature review and geospatial modelling exercise and step 3 is a structural assessment that will be conducted in all eligible facilities. Thus, sampling considerations only apply to steps 2, 4 and 5 of the study.

#### Step 2: usage—review of admission registers

All newborn admission registers (whether born within the facility, referred from another facility or brought from home) for a period of 1 year (July 2014 to July 2015) will be included in the usage and case-mix assessment. Additionally, maternal admission registers will be reviewed and information about residency and pregnancy outcome of women attending the facility to deliver will be obtained. Where facilities deliver 500 or fewer women per year, the register for a full year will be reviewed. Registers from those facilities that deliver more than 500 women during the sampling time frame will be sampled. A random list of weeks from the study period will be generated using Stata V.13. Starting from the top of this list, admissions from those weeks will be entered until a sample of 500 deliveries for that facility has been obtained. Although information is not available for all deliveries within potentially eligible facilities, experts advise that Pumwani Maternity Hospital (PMH) is the largest maternity hospital in Nairobi. Data from national statistics (District Health Information System 2) suggest annual deliveries of ∼25 000 per year. Hence, 500 deliveries at PMH would represent ∼2% of annual deliveries. In this scenario of the largest facility, with a sample of 500, we would be able to estimate a sample proportion (of residency and pregnancy outcome) of 50% with a 4.34% margin of error. This margin of error would be smaller for smaller facilities.

#### Step 4: process assessment—a nursing questionnaire

Nursing staff on duty at the time of a scheduled research team visit providing care to sick newborns or on the maternity ward will be invited to participate in a questionnaire at a time of their convenience and minimal disruption to care. Where more than three nurses are on-duty in the maternity ward or newborn unit, a random sample of half (rounded upwards) of the nurses from that ward and/or unit will be selected for interview. A list of all on-duty nurses will be made in alphabetical order of their names. Every odd numbered (ie, 1, 3, 5, etc) nurse from the list will be invited to participate. If a nurse declines to participate, then another nurse, starting from the top of the list (ie, 2, 4, etc), will be invited in their place.

#### Step 5: process assessment—a review of inpatient newborn case records

Across all facilities taking part in this study, we plan to sample 800 newborn case records. With a total population of 100 000 admissions (above which there is little change in sample size estimations), 800 records would estimate a 50% sample proportion (example outcomes include correct antibiotic dosage, correct fluid/feed dosage and route, evidence of clinical monitoring) with a margin of error of 3.45%. Given that the data generated from these reports will be clustered in facilities, we might expect a design effect, which will be accounted for during analysis. Allowing for a design effect of 2, a sample size of 800 records would estimate a 50% sample proportion with a margin of error of 4.89%.

### Measurements and outcomes

Quality of newborn care will be assessed by classifying it into the three components defined by Donabedian:^21^ (1) structure, characteristics of the setting in which care is administered, (2) process, the essential procedures in the delivery of care, and (3) outcome, the effect of care on the health status of the patient or population. The quality of care indicators measured in this study are summarised in table 1. Structural indicators and the nursing knowledge questionnaire score will be available for the maternity and neonatal units; all other indicators are only for the neonatal unit.

**Table 1:** Quality indicators to be assessed in the Nairobi Newborn Study

Structure	Process	Outcome
*(1) Infrastructure*: such as a clean water source, reliable electricity and a sink with soap for hand washing *(2) Equipment*: such as bag and mask, oxygen cylinder, nasal cannula/prongs, incubator, baby scale, cup to measure expressed breast milk, and intravenous fluid and infusion set *(3) Job aids*: such as feeding charts, fluids charts, Apgar charts, treatment sheets, and guidelines and protocols *(4) Essential drugs*: such as ampicillin, gentamicin, diazepam and dexamethasone *(5) Profile of human resources*: such as numbers of nurses, doctors and consultants	(1) Nursing knowledge questionnaire score* *(2) Frequency of performance of key neonatal interventions according to the nursing questionnaire (3) Adequacy of medical record documentation (4) Accuracy of prescription by clinicians:* for antibiotics, feeds and fluids *(5) Evidence of regular monitoring of vital signs*	*(1) Discharge outcome*: alive, dead, referred or absconded

*Structural indicators and the nursing knowledge questionnaire score will be available for the maternity and neonatal units. Other indicators are only for the neonatal unit.

### Descriptive statistics and main analyses

Study data will be collected and managed using REDCap electronic data capture tools hosted at the Kenya Medical Research Institute (KEMRI) Wellcome Trust Research Programme. REDCap is a secure, web-based application designed to support data capture for research studies. Data will be exported for cleaning and analyses in Stata V.13 (Stata Corporation).

#### Describing availability of healthcare for sick newborns

The availability of care for sick newborns in Nairobi City County will be described. Facilities deemed to provide inpatient newborn care will be mapped. This map will include information on the type, ownership, size, workload and level of care provided by each facility. The level of care will be described in terms of the structural capacity (ie, availability of staff, cots/beds, type of newborn unit—discrete or part of maternity, equipment and drugs to provide interventions) of the facilities. This information on structure will be used to categorise facilities into basic and comprehensive levels of neonatal care in consultation with the expert advisory group.

#### Indicators of quality of care

Quality of care indicators will be described to highlight key gaps in structure and process of neonatal care delivery in the maternity unit and neonatal inpatient unit across facilities. Variation in capacity and quality of care will be explored by health facility ownership: public/government, private, faith-based/not-for-profit.

Quality of care will be considered in the context of ongoing international and national efforts to define measurable neonatal health service indicators.^12^ ^22–25^ Most notably, findings from this study will be presented, where possible, in the context of the newborn and general indictors proposed by the WHO consultation on ‘Improving measurement of the quality of maternal, newborn and child care in health facilities’.^22^

#### Describe the gap between need and access

It is anticipated that there will be a shortfall in service provision, with some ill newborns not receiving the standard of care they require. We will formally explore this by comparing an estimation of need for care with the information we collect in this study on the access to, and usage of, an appropriate level of care.

The map of the magnitude and distribution of the burden of newborn conditions in Nairobi City County developed in step 1 of this study will be compared with information collected in step 2 about which patients are accessing and using care at facilities that have the ability to provide inpatient newborn care. The case-mix and numbers of patients attending these facilities will be compared with the estimated magnitude of the population-level burden of major neonatal conditions. Information about the residency of delivering women attending the health facility will be used to inform the estimated residency patterns of neonatal inpatients, therefore enabling a map of access to the facilities that provide inpatient newborn care to be created using geospatial mapping techniques. These facilities will be stratified by size and sector to enable estimates of service delivery patterns and geographic measures of access across the public, private and non-governmental sectors to be developed for Nairobi City County. This map of access will be compared with the map of burden of disease in order to provide details of the distribution of the gap and of areas within Nairobi City County with the greatest need for improved access to inpatient care for sick newborns.

Finally, we will explore approaches to estimating the possible number of lives saved by providing adequate access to inpatient care for sick newborns in Nairobi City County using internationally developed, open access modelling tools.^26^

## Dissemination

### Feedback to individual facilities

Feedback meetings will be held with facilities during which preliminary aggregate results will be presented. Facilities will have the option to request more specific feedback on their individual results for usage, resources and case record review. However, since knowledge is being assessed on a small number of nurses, specific feedback in this area will be withheld to protect the identity of nurses. Facilities will have the opportunity to ask questions and share thoughts with researchers about the findings and process of the project.

### Wider communication of findings

Opportunities will be available during the study to share progress updates and early findings with the project advisory group and, when preliminary results are available, with the Ministry of Health and other interested healthcare providers more widely. In particular, members of the project advisory group and investigators are already members of policymaking groups or forums such as the Inter-agency Coordinating Committee for Child and Newborn Health. Results of this study will be made more widely available through development of local reports provided to facilities and healthcare provider groups and subsequently presentation at local and international meetings and publication in peer-reviewed journals.

## Relevance to global public health

Under an ideal system, health service can be planned and monitored with the support of routine health information systems that provide comprehensive data from vital registration, health facility usage and cause of death notification.^12^ In settings such as Kenya, these data are lacking, making it difficult to plan service provision and measure quality of care. It is therefore important to augment any existing information systems with additional surveys. Partnering with local policymakers in conducting such research will ensure that the research is designed to answer locally relevant questions and the findings can be translated into meaningful policy and practice change.

This research will provide policymakers with vital information about the extent and quality of inpatient newborn services in Nairobi City County. Additionally, it will provide an estimation of shortfall in these services for meeting the expected demand. Such information will help to inform discussion with policymakers and stakeholders on how and where to improve services to promote equitable access. In particular, the outcomes of this study will facilitate the implementation of the recently published Kenya Essential Package for Health and Kenya Health Sector Referral Strategy, both of which are governmental strategic plans for 2014–2018.^23^ ^27^ Furthermore, these data will form the foundation of future work at a larger scale and studies planned to assess methods by which the delivery of neonatal care within health facilities can be improved.

Globally, there is an increasing recognition of the importance of neonatal health, leading to international efforts to recommend health service targets, quality indicators and measurement approaches.^4^ ^12^ ^22^ This study offers an approach to surveying provision and quality of neonatal health services within a resource-limited setting, across a whole population, engaging both public and private sectors. We hope that others will be able to draw on our experience and use our data collection tools in a combined effort to improve the care for this vulnerable population and make progress in reducing neonatal mortality.
